# A Deep Feature Fusion Underwater Image Enhancement Model Based on Perceptual Vision Swin Transformer

**DOI:** 10.3390/jimaging12010044

**Published:** 2026-01-14

**Authors:** Shasha Tian, Adisorn Sirikham, Jessada Konpang, Chuyang Wang

**Affiliations:** 1 Faculty of Engineering, Rajamangala University of Technology Krungthep, Bangkok 10120, Thailand; 669041810047@mail.rmutk.ac.th (S.T.); jessada.k@mail.rmutk.ac.th (J.K.); 2 School of Information Engineering, Jiangsu College of Finance and Accounting, Lianyungang 222061, China; 15805279538@163.com

**Keywords:** underwater image enhancement, U-shaped network, swim transformer, feature fusion

## Abstract

Underwater optical images are the primary carriers of underwater scene information, playing a crucial role in marine resource exploration, underwater environmental monitoring, and engineering inspection. However, wavelength-dependent absorption and scattering severely deteriorate underwater images, leading to reduced contrast, chromatic distortions, and loss of structural details. To address these issues, we propose a U-shaped underwater image enhancement framework that integrates Swin-Transformer blocks with lightweight attention and residual modules. A Dual-Window Multi-Head Self-Attention (DWMSA) in the bottleneck models long-range context while preserving fine local structure. A Global-Aware Attention Map (GAMP) adaptively re-weights channels and spatial locations to focus on severely degraded regions. A Feature-Augmentation Residual Network (FARN) stabilizes deep training and emphasizes texture and color fidelity. Trained with a combination of Charbonnier, perceptual, and edge losses, our method achieves state-of-the-art results in PSNR and SSIM, the lowest LPIPS, and improvements in UIQM and UCIQE on the UFO-120 and EUVP datasets, with average metrics of PSNR 29.5 dB, SSIM 0.94, LPIPS 0.17, UIQM 3.62, and UCIQE 0.59. Qualitative results show reduced color cast, restored contrast, and sharper details. Code, weights, and evaluation scripts will be released to support reproducibility.

## 1. Introduction

Underwater image enhancement (UIE) is crucial for marine observation, autonomous underwater vehicles, ecological monitoring, and subsea engineering [1,2,3]. However, obtaining reliable underwater imagery remains challenging due to wavelength-dependent absorption and scattering during light propagation in water [4]. These processes degrade images by suppressing contrast, introducing color casts, and blurring structural details, which hinders both scientific analysis and downstream computer vision tasks.

Existing UIE approaches can be broadly categorized into physics-based, non-physics-based, and deep-learning-based frameworks. Physics-based methods explicitly model the underwater image-formation process and attempt to invert the attenuation and scattering effects by estimating medium transmission and background light. While interpretable, such methods rely on idealized priors that generalize poorly to diverse underwater environments. Prior-based enhancement techniques—such as histogram equalization, Retinex decomposition, and fusion-based adjustments—modify pixel distributions to improve global visibility [5]. Nevertheless, their handcrafted assumptions often overlook spatially varying degradation and cross-channel attenuation, leading to color distortions or over-enhancement in complex scenes.

Recent deep learning-based UIE models have demonstrated superior adaptability by learning degradation patterns directly from data. CNN-based methods capture local spatial structures but struggle to handle long-range dependencies, resulting in incomplete correction of globally attenuated regions [6]. GAN-based frameworks enhance perceptual realism but suffer from training instability and may introduce hallucinated textures [7]. More recently, Transformer architectures and Swin Transformer variants have demonstrated the ability to model global context. However, directly applying these models to underwater imagery is suboptimal: local high-frequency textures are under-modeled, channel-wise degradation is treated uniformly, and spatially uneven attenuation is not explicitly addressed.

To address these limitations, we propose a perception-guided deep feature fusion framework based on a U-shaped Swin Transformer architecture for underwater image enhancement. Our main contributions of this work can be summarized as follows:

- We propose a perception-guided U-shaped Swin Transformer framework to model hierarchical representations for underwater image enhancement. The proposed framework explicitly addresses the spatially non-uniform and spectrally dependent attenuation inherent in underwater imagery.
- We introduce a Global-Aware Attention Map (GAMP) to emphasize attenuated color channels and spatial regions. GAMP jointly models multi-scale spatial degradation and channel-wise attenuation to guide degradation-aware feature modulation.
- We develop a Dual-Window Multi-Head Self-Attention (DWMSA) that integrates small-window and overlapping-window attention, unifying global context modeling with fine-grained texture preservation.
- We design a Feature-Augmentation Residual Network (FARN) to stabilize deep optimization and enhance the recovery of high-frequency details and chromatic fidelity across diverse underwater conditions.

## 2. Related Works

UIE aims to improve the perceptual and analytical quality of underwater imagery, and existing methodologies can be broadly categorized into three paradigms: physics-based models, non-physics-based models, and deep learning-based models.

### 2.1. Physics-Based Models

Physics-based approaches formulate underwater image enhancement as an inverse problem grounded in optical image formation models, where wavelength-dependent absorption and scattering are explicitly modeled. Typical techniques include dark-channel-based restoration, depth-dependent attenuation estimation, and wavelength compensation strategies.

The first leverages the Dark Channel Prior (DCP) [8] for parameter refinement. Representative techniques include the integration of DCP with wavelength-specific compensation strategies for color restoration, as well as the Underwater Dark Channel Prior (UDCP).

The second subclass exploits inherent optical characteristics of underwater imaging to infer relationships among channel-wise attenuation, background light, and water body properties. Li et al. [9] proposed methods grounded in histogram priors and minimal information-loss constraints; Peng [10] jointly modeled image blur and absorption to estimate scene depth. Despite their interpretability, these approaches impose practical constraints, often requiring multiple overlapping views, accurate camera calibration, or auxiliary structure-from-motion pipelines such as COLMAP [11]. Moreover, physics-based models lack the ability to dynamically reason about spatial–channel degradation patterns, limiting their applicability to diverse underwater scenes.

### 2.2. Non-Physics-Based Models

Non-physics-based methods avoid explicit optical models, instead relying on statistical or perceptual priors such as histogram redistribution, Retinex-based decomposition, and fusion strategies. Examples include Iqbal et al.’s RGB/HSV dynamic-range stretching [12], Ghani’s Rayleigh-distribution-based adjustment to suppress over-enhancement [13], and Akkaynak et al.’s adaptive multi-interval sub-histogram equalization scheme [14].

A second influential line of work builds upon Retinex theory, which decomposes an image into illumination and reflectance components. Fu et al. [15] introduced a three-stage pipeline involving color correction, layer decomposition, and enhancement; Zhou et al. [15,16] incorporated pixel-distribution remapping with multi-prior variational constraints for efficient decoupling; Wang et al. [17] employed an improved multi-scale Retinex for the V-channel in HSV space and applied detail fusion via guided filtering; and Jiang et al. [18] proposed a dual-path Mutual Retinex framework that utilizes mutual learning to more accurately separate illumination and reflectance.

These approaches can improve global contrast and color balance, yet their hand-crafted assumptions make them sensitive to noise and illumination variations [19]. In particular, they struggle with region-wise attenuation differences and often introduce over-enhancement artifacts in turbid waters [20]. Their limited capacity for structural modeling restricts their applicability for fine-detail preservation required in modern underwater vision tasks.

### 2.3. Deep Learning-Based Models

UIE has long been a topic of sustained attention within the computer-vision community, yielding numerous influential contributions [21,22,23]. More recently, deep-learning-based approaches have emerged as the dominant methodology, with current research primarily centered around three architectural families: CNNs, GANs, and Transformer-based models.

CNN–based approaches have been widely used in UIE, as their hierarchical filtering enables the extraction of discriminative features. Zhang et al. [24] presented a residual multi-scale CNN that integrates receptive fields of varying sizes to model degradation behaviors more comprehensively. Chen et al. [25] proposed CASF-Net, which fuses residual learning with spatial–channel feature integration to stabilize training while delivering accurate color restoration. However, the local receptive field of standard convolutions hinders the modeling of long-range dependencies, which is crucial for correcting large-scale color casts and uneven attenuation prevalent in underwater scenes.

Generative adversarial networks (GANs) have been applied to underwater image enhancement, leveraging adversarial learning between a generator and a discriminator to improve perceptual realism. Deng et al. [26] introduced UCT-GAN, which learns an inverse color-mapping function to counteract underwater color attenuation, achieving effective restoration under limited training data. Guan et al. [27] proposed AUIE-GAN, integrating self-attention within the generator to capture long-range dependencies and employing deep residual blocks to mitigate gradient instability. Mu-GAN [28] further combines convolutional processing with attention mechanisms and utilizes dual discriminators alongside a multi-objective loss to enhance both chromatic accuracy and perceptual realism. However, GAN training is notoriously unstable, and the adversarial process can introduce unrealistic textures or artifacts, compromising the faithfulness of the enhanced image.

The Swin Transformer is a hierarchical vision transformer that performs self-attention within local non-overlapping windows, resulting in linear computational complexity with respect to image size. By adopting a shifted window strategy between consecutive layers, it enables effective information exchange across neighboring windows while preserving local structural representations. This design allows the Swin Transformer to jointly capture local textures and long-range contextual dependencies, making it particularly suitable for dense vision tasks such as underwater image enhancement. Transformer-based architectures have been introduced for UIE due to their ability to model long-range dependencies and global contextual interactions [29,30,31,32]. Zhang et al. [33] proposed U-TWGAN, which integrates wavelet decomposition with hierarchical Transformer modeling to achieve effective image enhancement while maintaining computational efficiency. Liu et al. [34] proposed a hierarchical Transformer that sequentially performs structural enhancement followed by detail refinement, thereby ensuring both global consistency and local fidelity. To improve training scalability, He et al. [35] developed the masked autoencoder (MAE), which employs random patch masking and an asymmetric encoder–decoder design, improving representational accuracy for underwater imagery.

Despite these advances, Transformer-based and hybrid architectures still exhibit several inherent limitations in underwater image enhancement. First, they often show limited degradation awareness due to the absence of explicit mechanisms for modeling spatial–channel attenuation characteristics. Second, the sequential alternation of window-based and shifted-window self-attention restricts effective global–local feature interaction, thereby constraining cross-window information exchange. Finally, deep self-attention operations tend to over smooth textures by suppressing high-frequency components, which are crucial for faithful reconstruction of fine underwater details.

In summary, current UIE approaches do not jointly address spatial–channel degradation perception, efficient global–local context modeling, and texture-fidelity preservation within a unified framework. To overcome these limitations, we propose a perceptual vision Swin-Transformer architecture that integrates a GAMP module for degradation-aware feature refinement, a DWMSA module for parallel global–local attention fusion, and a FARN module for restoring high-frequency textures and chromatic accuracy. This design directly targets long-standing challenges in underwater imaging, providing a principled and comprehensive solution.

## 3. Methodology

We propose a U-shaped underwater image enhancement network, termed the Perceptual Vision Swin Transformer, which integrates novel attention and feature refinement modules within a Swin Transformer backbone. To articulate the underlying design rationale, this section first outlines the overall architecture and subsequently provides detailed descriptions of the GAMP module, the DWMSA module, and the FARN module.

### 3.1. Model Architecture

**Figure 1:**
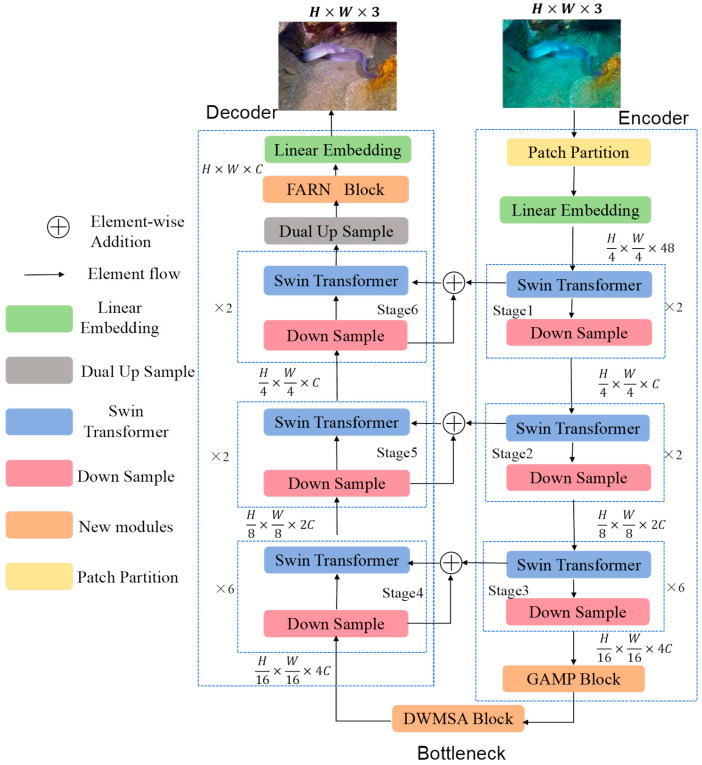
Overall Perceptual Vision Swin Transformer-Based Deep Feature Fusion UIE Model.

The overall architecture follows a U-shaped design, as illustrated in Figure 1. The encoder hierarchically extracts multi-scale representations while retaining local textures and structural cues. Building on these features, the GAMP module performs global and spatially aware attenuation correction to produce degradation-adaptive feature refinements. At the network bottleneck, the DWMSA module jointly models long-range dependencies and localized contextual interactions to improve consistency between global structure and regional details. Finally, the decoder progressively reconstructs spatial resolution and integrates the FARN module to further strengthen fine-grained texture fidelity, yielding improved visual restoration.

#### 3.1.1. Encoding Module

The encoder consists of Patch Partitioning, Linear Embedding, three hierarchical representation stages, and the GAMP module. Patch Partition extracts non-overlapping 4 × 4 patches, reducing redundancy while preserving localized structure. Linear Embedding then projects each patch into a 48-dimensional feature vector, yielding an initial representation of size $\frac{H}{4}\times \frac{W}{4}\times 48$. The hierarchical stages process features as follows:

Stage 1: Two Swin Transformer blocks to model short-range interactions via window attention.

Stage 2: Two blocks with doubled channel capacity 2*C* and reduced spatial resolution $\frac{H}{8}\times \frac{W}{8}$

Stage 3: Six blocks capturing longer-range dependencies essential for complex underwater degradations, outputting features of size $\frac{H}{16}\times \frac{W}{16}\times 4C$.

The GAMP module then reweights spatial regions and color channels most affected by attenuation, suppressing irrelevant responses and providing refined features to the bottleneck.

#### 3.1.2. Bottleneck Module

The original W-MSA/SW-MSA alternation fuses cross-window information only in a sequential manner, resulting in limited global–local interaction for underwater texture modeling. Our dual-window design overcomes this by processing fine-grained local patterns and broader contextual dependencies in parallel. The incoming feature map $\frac{H}{16}\times \frac{W}{16}\times 4C$ is divided into non-overlapping windows. DWMSA computes attention along two complementary paths: small-window attention for extracting fine-grained local patterns and overlapping-window attention for capturing broader contextual dependencies. After layer normalization and generation of *Q*, *K*, and *V* through 1 × 1 convolutions, attention scores are obtained using relative position bias. The outputs from both paths are fused, producing representations that integrate global dependencies with spatial structure.

#### 3.1.3. Decoder Module

The decoder reconstructs high-resolution features via three hierarchical stages, Dual Up Sample operations [36], Linear Embedding projection, and the FARN.

Stage 4: Performs semantic-level decoding and upsamples resolution to $\;\frac{H}{8}\times \frac{W}{8}$.

Stage 5: Restores mid-scale features and increases resolution to $\frac{H}{4}\times \frac{W}{4}$.

Stage 6: Focuses on fine-detail recovery, producing feature maps of size H×W×48.

Skip connections merge encoder and decoder features at corresponding levels, allowing low-level structural cues and high-level contextual information to be propagated jointly.

The Linear Embedding module then projects the features back to RGB space, while the FARN block stabilizes training through residual learning and enhances texture integrity and chromatic accuracy. The final enhanced output is of size *H* × *W* × 3.

### 3.2. GAMP

**Figure 2:**
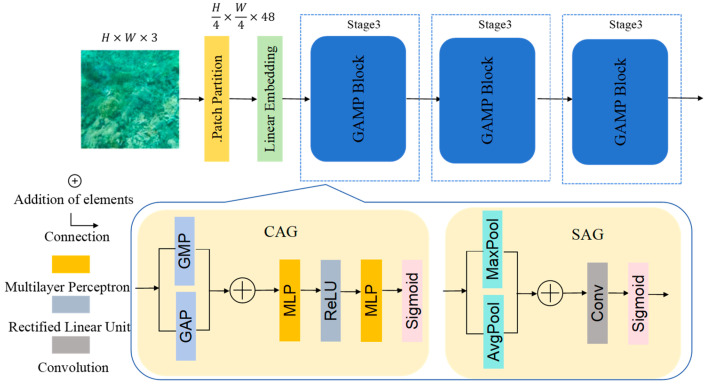
Overall architecture of GAMP.

Underwater illumination decays unevenly across spectral channels and spatial regions, often leading to pronounced color shifts and brightness inconsistencies. To overcome attenuation patterns that conventional channels have, we introduce the GAMP module—a degradation-aware unit positioned at the encoder output. The structure of GAMP is shown in Figure 2. Within GAMP, a Channel Attention Generation (CAG) is employed to adaptively reweight channel-wise feature responses based on their global importance, whereas a Spatial Attention Generation (SAG) focuses on highlighting informative spatial regions by modeling spatial attention maps.

#### 3.2.1. Multi-Scale Degradation Feature Extraction

By assigning adaptive spatial and channel-wise weights, GAMP emphasizes severely degraded regions and produces more discriminative representations.

Given an input feature map $F\in R^{H\times W\times C}$, multi-scale degradation features are extracted using parallel convolutional branches with different kernel sizes, defined as

(1) $$D_{i}={Conv}_{k_{i}}\left(F\right),\;\;\;\;\;\;i\;\in \;\left\{1,\;2,\;3\right\},\;\;k_{i}\;\in \;\{1,\;3,\;5\}$$

where $D_{i}$ denote feature maps capturing degradation characteristics at different spatial scales.

The features extracted from all branches are then fused through channel-wise concatenation followed by a 1 × 1 convolution:

(2) $$F_{d}={Conv}_{1\times 1}(Concat(D_{1},\;D_{2},\;D_{3}))$$

where the 1 × 1 convolution is used to adaptively integrate multi-scale information and reduce channel redundancy.

#### 3.2.2. Channel Attention Generation

Global statistical information is extracted using Global Average Pooling (GAP) and Global Max Pooling (GMP), which capture complementary global context by aggregating feature responses across spatial dimensions. GAP summarizes the mean activation of each channel, providing a global descriptor of overall feature distribution, while GMP emphasizes the most salient responses by selecting the maximum activation. These pooled features are subsequently fed into the attention generation process to enhance global awareness. The channel attention focuses on global degradation across channels using GAP and GMP, which are first combined and then passed through an MLP with a squeeze ratio of *r* = 16:

(3) $$A_{c}=\sigma (W_{2}(\delta (W_{1}(GAP\left(F_{d}\right)+GMP(F_{d})))))$$

where $W_{1}\in R^{C/r\times C}$ and $W_{2}\in R^{C\times C/r}$ are learnable weights, δ(.)  is the ReLU function, and σ(.) is the Sigmoid function. The resulting $A_{c}\in R^{C\times 1\times 1}$ adaptively weights the feature channels according to the degree of degradation.

#### 3.2.3. Spatial Attention Generation

After channel refinement, the spatial attention mechanism accurately captures locally degraded regions. This mechanism fuses the average-pooled and max-pooled features along the channel dimension and applies a 7 × 7 convolution operation, as described below.

(4) $$A_{s}=\sigma ({Conv}_{7\times 7}([AvgPool(F_{c});\;MaxPool(F_{c})]))$$

where [;] denotes channel-wise concatenation. The spatial attention $\;A_{s}\in R^{1\times H\times W}$ emphasizes the most degraded areas in the feature space.

Finally, both attention maps are combined to refine the feature representation:

(5) $$F_{GAMP}=F_{d}\times A_{c}+F_{d}\times A_{s}$$

This approach focuses on heavily degraded regions, which refer to spatial areas severely affected by underwater-specific degradations such as strong light absorption, scattering, color attenuation, and low signal-to-noise ratio. These regions typically exhibit reduced contrast, color distortion, and loss of fine structural details.

By adaptively reweighting feature responses according to the degradation severity, the network allocates more representational capacity to these challenging areas. This selective emphasis reduces unnecessary feature amplification in well-preserved regions, leading to smoother gradient propagation and more stable optimization during training.

### 3.3. DWMSA

**Figure 3:**
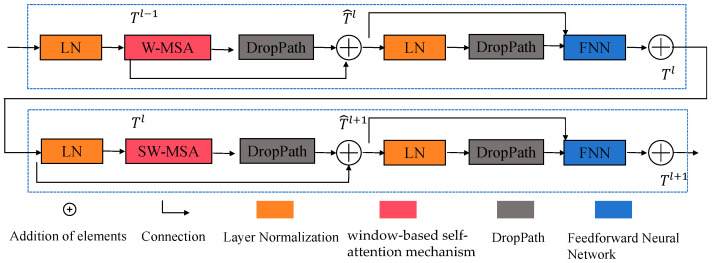
Overall architecture of DWMSA.

The DWMSA module extends the conventional window-based multi-head self-attention (W-MSA) mechanism [37]. Compared with global self-attention, W-MSA reduces computational complexity while preserving local structural information, which is particularly important for underwater image enhancement where degradations are spatially variant. The structure of DWMSA is shown in Figure 3. Standard window-based attention effectively reduces computational cost but limits cross-window interactions, hindering the modeling of long-range dependencies in underwater scenes. To overcome this limitation, DWMSA combines two complementary attention mechanisms: (1) small-window attention, which captures fine-level textures, and (2) overlapping-window attention, which enables cross-region information exchange.

In addition, a DropPath operation is applied as a stochastic depth regularization technique during training. By randomly dropping residual paths with a certain probability, DropPath effectively mitigates overfitting and improves model generalization without introducing additional inference-time overhead. This design helps stabilize the training of deep transformer blocks and enhances robustness under complex underwater conditions.

The feature information obtained from the coding layer is input into DWMSA for secondary processing. This can be formulated as follows, in which the feature output is represented by DWMSA, the output feature represents the FNN module, LN denotes the Layer Normalization layer, and FNN refers to the Feedforward Neural Network [38]. The computation process is defined by Equations (6)–(9):

(6) $${\hat{T}}^{l}=W-MSA\left(LN\left(T^{l-1}\right)\right)+T^{l-1}$$

(7) $$T^{l}=FNN\left(LN\left({\hat{T}}^{l}\right)\right)+{\hat{T}}^{l}$$

(8) $$T^{l}=SW-MSA\left(LN\left(T^{l}\right)\right)+T^{l}$$

(9) $$\;\;\;\;\;\;\;\;\;T^{l+1}=FNN\left(LN\left({\hat{T}}^{l+1}\right)\right)+{\hat{T}}^{l+1}$$

${\hat{T}}^{l}$ represents the output of W-MSA, and $T^{l}$ represents the output of the FNN module.

The computation process of DWMSA starts from a feature map, which is divided into multiple non-overlapping windows. Suppose the input feature map has a size of h×w×C, and the size of each window is M×M, the feature map is separated into $\frac{h}{M}\times \frac{w}{M}$ windows. The features within each window are processed by Layer Normalization (LN) to stabilize the model’s training. The normalized features are then fed into the DWMSA module, where they pass through convolution layers and Linear Projection (LP).

The convolution layers are used to capture local spatial information. By sliding different convolution kernels within the window, local features are extracted. The convolution layers generate the Query (*Q*), Key (*K*), and Value (*V*) vectors, which are used in the subsequent self-attention computation.

The *Q*, *K*, and *V* tensors are generated through linear projection layers, implemented as 1 × 1 convolutions that perform a learned linear combination across the channel dimension at each spatial location. Specifically, for an input feature map $F\in R^{H*W*C}$

(10) $$Q=FW_{Q},\;K=FW_{K},\;V=FW_{V}$$

where $W_{Q}$,${\;W}_{K},{\;W}_{V}\in R^{C*d}$, and the resulting tensors have shapes $Q,\;K,\;V\in R^{(H*W)*d}$.

Linear Projection maps the input features to three different subspaces through weight matrices corresponding to *Q*, *K*, and *V*. The self-attention is computed using a relative position bias *B*, according to the formula presented below:

(11) $$Attention\left(Q,\;K,\;V\right)\;=\;Softmax\left(\frac{QK^{T}}{\sqrt{C}}+B\right)V$$

Here, $QK^{T}$ calculates the similarity between the query vector *Q* and the key vector *K* to obtain attention scores; $\frac{1}{\sqrt{C}}$ is a scaling factor to prevent large attention values; *B* is the Relative Position Bias (RPB), which considers the relative positional relationships within a window. The Softmax function can normalize the attention scores to determine the attention score for each position. Finally, it uses the attention scores to fuse the value vector *V* to obtain the self-attention output within each window.

(12) $$Attention={Conv}_{1\times 1}(D\_Conv(DW\_Conv(F)))$$

(13) out=F×Attention

where *F* represents the input feature, DS_Conv is the depthwise separable convolution, and D_Conv is the dilated convolution. The formula for the crossfusion module is shown below. Here, the crossfusion module refers to a feature interaction block that enables bidirectional information exchange between parallel feature streams by jointly integrating complementary representations from different branches.

(14) $$F=Cat(GELU({Conv}_{1\times 1}(F^{T}),\;GELU({Conv}_{1\times 1}(F^{A})))$$

(15) $$F_{D}=Downsample(GELU({Conv}_{3\times 3}(F)))$$

(16) $$F_{DWMSA}=F_{D}+F$$

Here, $F^{T}$ denotes the feature representation produced by the Transformer, corresponding to the small-window configuration. ${\;F}^{A}$ denotes the feature representation generated by the attention module, corresponding to the overlapping-window configuration. The feature information is passed to the decoder before downsampling. In Equation (15), the downsampling operation is implemented using a strided convolution, which simultaneously reduces the spatial resolution and increases the receptive field.

In the DWMSA module, ${Conv}_{1*1}$ represents the operation in which the two input feature maps are first compressed using a 1 × 1 convolution for dimensionality reduction, after which the reduced features are concatenated and subsequently fused through a 3 × 3 convolution. In this way, the two branches are able to exchange complementary information while simultaneously extracting distinct feature representations. The proposed DWMSA module not only compensates for the performance degradation of the original Swin Transformer on small-scale datasets but also enables more efficient localization of structurally important regions within the feature space.

### 3.4. FARN

**Figure 4:**
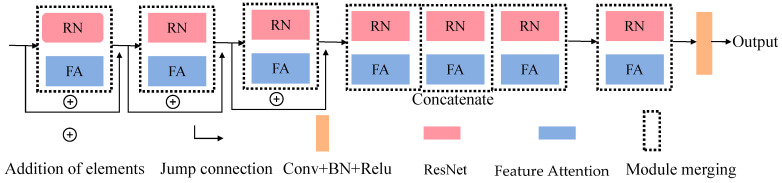
Overall architecture of FARN.

To enhance texture fidelity and stabilize optimization in deeper layers, we develop a FARN. Each FARN block consists of a residual convolutional unit coupled with a lightweight feature-attention module. Each FARN block adopts a ResNet-based residual unit (3 consecutive convolutional layers) to stabilize gradient propagation, combined with a feature-attention module for adaptive feature refinement. The overall architecture of FARN is shown in Figure 4.

#### 3.4.1. Channel Attention

After global modeling by the Transformer, feature representations often become overly smoothed, leading to the loss of fine textures. Since the decoder requires strong texture priors when progressively restoring spatial resolution, we introduce the FARN module to reinforce texture fidelity.

The feature attention model consists of channel attention (CA) and pixel attention (PA). Specifically, CA’s core function is to establish correlations between channels, assigning equal weights to all spatial locations within each channel; PA, on the other hand, assigns weights corresponding to all channels to each specific spatial location. This dual design allows the network to emphasize informative channels and spatial regions, such as edges or blurred areas. Given an input feature map $F\in R^{C\times H\times W}$, the corresponding calculation is as follows:

(17) $$g_{c}=H_{p}\left(F_{c}\right)=\frac{1}{H\times W}\sum_{i=1}^{H}\sum_{j=1}^{W}X_{c}(i,j)$$

where $F_{c}$ refers to the c-th channel feature map of the input image, $X_{c}(i,j)$ represents the pixel value at position (i,j) in channel $X_{c}$, and $H_{p}$ denotes the global pooling function. $g_{c}\in R^{C}$ represents the aggregated channel descriptor. The size of the feature map is changed from C×H×W to C×1×1. To obtain the attention weights for each channel, the aggregated feature is passed through two convolution layers with a squeeze ratio of *r* = 16 to reduce computational cost and improve generalization. It is followed by a ReLU and a Sigmoid activation function. The channel attention is then applied to the input via element-wise multiplication, as formulated below:

(18) $$CA_{c}=\sigma ({\mathrm{Conv}}_{1\times 1}^{up}(\delta ({\mathrm{Conv}}_{1\times 1}^{down}(g_{c}))))$$

(19) $$F_{c}^{*}=CA_{c}⊗F_{c}$$

Here, $\sigma \left(.\right)\;\;and$ δ(.) denote the Sigmoid and ReLU activation function, respectively, and the channel dimension is reduced from C to C/r and then restored to C. $CA_{c}$ is the attention weight for the c-th channel.

#### 3.4.2. Pixel Attention

Given the non-uniform distribution of color deviations across pixels in underwater images, a Pixel Attention module is employed to allow the network to focus more on informative spatial features. The channel-refined feature $F_{c}^{*}$ is processed through two convolutional layers with ReLU and Sigmoid activations to generate a spatial attention map:

(20) $$PA=\sigma ({\mathrm{Conv}}_{3\times 3}^{2}(\delta ({\mathrm{Conv}}_{3\times 3}^{1}(F_{c}^{*}))))$$

(21) $$FA=F_{c}^{*}⊗PA$$

In Equation (21), the symbol ⊗ denotes element-wise multiplication between the attention map and the corresponding feature map, which is used to modulate feature responses according to learned attention weights.

Finally, residual fusion preserves original structures and prevents over-enhancement, where over-enhancement refers to excessive amplification of brightness or contrast that may lead to visual artifacts such as color distortion, saturation, or loss of natural appearance.

(22) $$F_{FARN}=FA+F$$

The residual network and feature attention mechanism of the FARN module complement each other. The residual structure captures semantic features, while the FA mechanism can distinguish different features across channels and spatial locations.

## 4. Experiments and Results Analysis

### 4.1. Experimental Preparation

The proposed model was developed in Python 3.10.13 using the PyTorch 2.1.0 framework and trained on a workstation equipped with an NVIDIA GeForce RTX 4060 GPU (NVIDIA Corporation, Santa Clara, CA, USA), 32 GB of system memory, and CUDA 11.8 support. We utilized cuDNN, NVIDIA’s CUDA Deep Neural Network library, to accelerate deep learning computations on the GPU, thereby improving training efficiency. Optimization was performed using Adam with a learning rate of 1 × 10^−4^ and a batch size of 8. The experimental and implementation parameters are presented in Table 1.

#### 4.1.1. Dataset Selection

We validated the performance of our method on three representative public UIE datasets: UFO-120 [39], EUVP [40] and UIEB. To ensure the fairness and comparability of the experiments, all evaluations were conducted following the scene-distribution standards of each dataset’s official test set. We then evaluated the enhancement effect of each model from both subjective and objective evaluation metrics.

The UFO-120 dataset contains 1620 paired images, consisting of 1500 images officially provided for training/testing, and 120 images officially provided for validation/testing. These images cover a wide range of underwater scenes, including marine life, seabed topography, and artificial structures.

The EUVP dataset contains 2185 paired annotated images, which are divided into three subsets with different scenes (nearshore shallow water, offshore deep water, and complex biological areas). To ensure fairness of experiments and balance in sample distribution, we used 800 training images, 300 validation test images with reference ground truth. This experimental subset was not directly taken from the official full split, but was randomly sampled in proportion from the three official subsets.

In addition, we use the UIEB dataset, which contains 890 real-world underwater images along with corresponding high-quality reference images for quantitative evaluation. We further employ the UIEB-90 subset, consisting of 90 test images without reference ground truth, which is used exclusively for qualitative visual comparison. Together, these datasets support the evaluation of the model’s generalization ability and robustness.

It is worth noting that, due to the complexity of underwater environments, obtaining true ground truth (GT) images for underwater scenes is challenging. Therefore, the GT images provided in benchmark datasets are not physically captured true GTs. Instead, they are reference images generated through basic enhancement methods and selected through expert consensus or evaluation to represent visually optimal results. While these reference-based GTs enable effective experimental comparison, they may also introduce potential subjective bias. These datasets enable consistent performance comparison and evaluation of generalization.

#### 4.1.2. Selection of Metrics

To evaluate the model’s performance, Learning Perceived Image Block Similarity (LPIPS), Peak Signal-to-Noise Ratio (PSNR), Structural Similarity Index Measure (SSIM) [41], Underwater Image Quality Measure (UIQM) [42], and Underwater Color Image Quality Evaluation (UCIQE) [43] were selected as evaluation metrics.

LPIPS measures perceptual distance, where lower values indicate higher perceptual consistency. PSNR is a distortion-based metric. Higher PSNR values generally correspond to lower reconstruction error. SSIM assesses structural similarity by jointly considering luminance, contrast, and structural information, and has been used to evaluate perceptual similarity between images.

For non-reference evaluation, UIQM is adopted as an underwater-specific image quality metric. Given that color distortion, blurring, and low contrast are prevalent in underwater imagery, UIQM evaluates enhancement quality under underwater imaging conditions. In addition, UCIQE evaluates underwater image quality based on colorfulness, luminance, and contrast, serving as a perceptually motivated metric related to human visual perception.

### 4.2. Simulated Training

The proposed network is trained end-to-end using the Adam optimizer, which provides stable optimization with moderate computational cost. A mini-batch size of 8 is adopted, with each iteration processing eight randomly sampled image–label pairs.

All experiments are conducted with a fixed random seed of 42 to ensure reproducibility. A linear warm-up strategy is applied for the first five epochs, increasing the learning rate from 1 × 10^−5^ to 1 × 10^−4^ to stabilize early training. In addition, an L2 weight decay of 1 × 10^−4^ is applied to regularize the optimization process and reduce overfitting. The base learning rate is set to 1 × 10^−4^. Ablation studies indicate that this learning-rate configuration maintains stable training dynamics. The network is trained for 400 epochs, which provides stable optimization and consistent performance on both training and validation sets.

For the loss function calculation, the model uses a weighted sum of three losses. The Charbonnier loss [44] is a variant of the L1 loss that introduces a constant factor ε to smooth the gradient and resolve instability issues in gradient computation. Its formula is

(23) $$L_{Ch}\left(x,y\right)=E_{x-p(r),y-p(g)}\sqrt{(x-y{)}^{2}+\varepsilon^{2}}$$

where x represents the enhanced image, y represents the ground truth image, and the constant ε is set to $5\times 10^{-3}$.

The perceptual loss [45] in this study is defined as the difference in feature representations between the generated image and the original image. These representations capture both semantic and structural information. A pre-trained VGG19 network is used to extract feature maps, and these features are employed to supervise the generated ones, enabling the model to learn high-level perceptual cues. Its formula is

(24) $$L_{Per}={\left||\Phi \left(y\right)-\Phi (x)|\right|}_{2}v$$

Here, y represents the original image, and x represents the generated image. *Φ*(⋅) denotes the feature representations extracted from the pre-trained VGG19 network. To enhance high-frequency edge details, an edge loss focusing on gradient information is introduced, defined as

(25) $$L_{\mathrm{Edge}}\left(x,y\right)=\sqrt{\|\Delta x-\Delta y{\|}^{2}+\varepsilon^{2}}$$

where Δ(∗) denotes the Laplacian operator.

(26) $$L_{\mathrm{sum}}=\lambda_{1}L_{Ch}+\lambda_{2}L_{\mathrm{Per}}+\lambda_{3}L_{Edge}$$

In the above equation, $\lambda_{1},{\;\lambda }_{2},{\;\lambda }_{3}$ are balancing parameters that represent the weights of each loss term. In this study, after extensive training and parameter tuning, they are finally set to $\lambda_{1}=\;$0.3, $\lambda_{2}=\;$0.5, and $\lambda_{3}=\;$0.2, respectively. By adjusting these parameters, the model can comprehensively consider different aspects of the image to balance multiple image quality factors during training.

### 4.3. Experimental Results Analysis

#### 4.3.1. Subjective Analysis and Comparison

While several recent transformer-based UIE methods have been proposed, many of these approaches do not yet provide publicly available and reproducible implementations, or adopt substantially different training datasets and evaluation protocols. To ensure a fair and consistent comparison under unified experimental settings, we therefore restrict our quantitative comparisons to representative and widely adopted baseline methods. We evaluated our perceptual Swin Transformer model by comparing it with several representative UIE algorithms, including UWCNN [46], UGAN [47], FUnIE_GAN [32], WaterNet [48], Deep_SESR [31], and URSCT_SESR [49].

**Figure 5:**
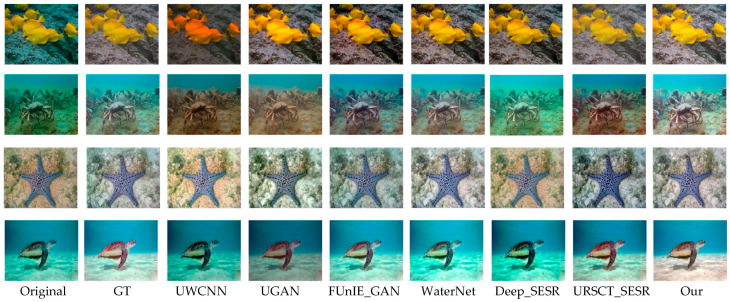
Subjective evaluation of various models of UFO-120.

**Figure 6:**
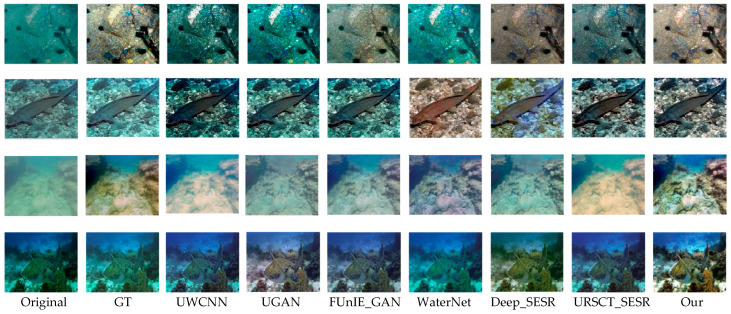
Subjective evaluation of various models of EUVP.

**Figure 7:**
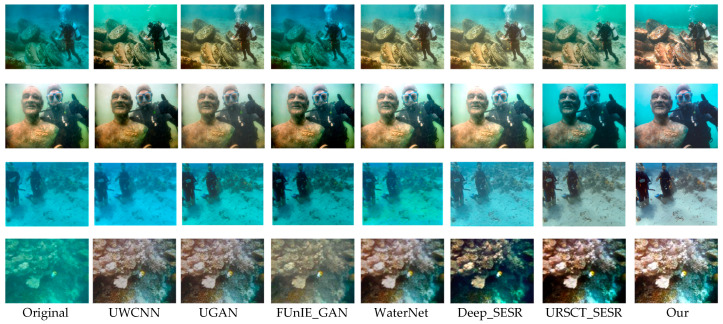
Subjective evaluation of various models of UIEB-90.

To provide an intuitive assessment of the proposed framework, we benchmark it against a set of representative state-of-the-art underwater image enhancement methods across three widely used datasets—UFO-120, EUVP, and UIEB-90. Representative qualitative results are presented in Figure 5, Figure 6 and Figure 7.

We first examine performance on datasets that include reference GT images. As shown in Figure 5, several existing methods suffer from characteristic limitations. UWCNN tends to introduce a persistent greenish color cast and blurred object boundaries. UGAN produces images with reduced brightness and desaturated appearance, leading to visually unrealistic results. FUnIE-GAN and Deep_SESR alleviate some color distortion but still exhibit noticeable chromatic shifts and insufficient tonal richness. WaterNet struggles to maintain stable color balance across different regions, while URSCT-SESR, despite partially suppressing the blue–green bias, fails to fully restore saturation and contrast. In contrast, the proposed method generates images with more natural color distribution, clearer structural details, and improved visual consistency.

As shown in Figure 6, similar performance trends are observed on the EUVP dataset. UWCNN produces overly dark images with visible artifacts; UGAN and Deep_SESR often generate visually implausible enhancements; FUnIE-GAN remains susceptible to severe color casts; and WaterNet introduces appreciable texture loss and degraded sharpness. URSCT_SESR continues to exhibit residual color bias. In contrast, the proposed model produces images with richer tonal gradients, well-balanced saturation, and sharper object contours and textures, achieving improved perceptual quality across diverse scenes.

As shown in Figure 7, we then evaluate real-world samples lacking reference GTs. FUNIE-GAN exhibits substantial inaccuracies in recovering coral coloration; UWCNN demonstrates limited generalization due to its lightweight architecture; FUR-SCT_SESR and WaterNet introduce distinct greenish artifacts; and Deep_SESR fails to completely remove haze in several cases. Other competing models display scene-dependent distortions. By comparison, the proposed approach effectively corrects chromatic distortions while preserving fine-scale structural details, delivering stable perceptual quality across diverse underwater conditions.

In summary, conventional enhancement-based methods tend to over-amplify brightness or introduce noticeable color bias, while GAN-based approaches often suffer from artificial textures and spatially inconsistent illumination. CNN-based models recover local details well, but their limited receptive fields make global color correction less effective. By contrast, the proposed method achieves clearer structural representation, more accurate color rendition, and improved illumination stability, benefiting from the complementary integration of GAMP, DWMSA, and FARN. Fine-grained details, such as coral textures and object boundaries, are reconstructed, producing consistent enhancement results across complex underwater scenes.

Table 2 reports the average subjective visual quality scores evaluated by 50 human observers. The observers were graduate students and researchers with normal vision. All images were displayed on the same calibrated monitor under consistent indoor lighting conditions. The presentation order of images and methods was randomized to avoid bias. Before the evaluation, the observers were instructed to rate each enhanced image on a 1–10 scale, where higher scores indicate better overall visual quality, taking into account color fidelity, contrast, and structural clarity. Consistent with the qualitative comparisons, conventional enhancement-based methods receive lower scores due to underexposure, color bias, or visual artifacts. GAN-based approaches show moderate improvement but remain affected by perceptual inconsistency. In contrast, our method achieves the highest mean subjective score among all compared approaches, indicating superior perceptual quality in terms of color fidelity, structural clarity, and overall visual consistency.

**Figure 8:**
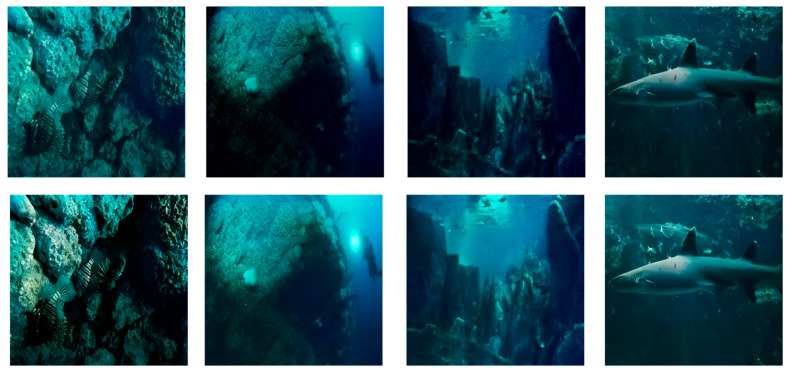
Failure Cases.

Despite its overall effectiveness, the proposed model does not generalize equally well across all underwater conditions. As illustrated in Figure 8, representative failure cases occur in extremely low-light scenarios, where severe light absorption and scattering significantly suppress global illumination, obscure fine structural details, and induce pronounced color distortion.

This degradation can be attributed to two primary factors. First, under extremely low illumination, the signal-to-noise ratio of the input images is substantially reduced, causing high-frequency details to be dominated by noise rather than meaningful structural information. As a result, feature representations extracted by both convolutional and attention-based modules become less discriminative. Second, the illumination enhancement process in such scenarios tends to amplify noise together with brightness, making it difficult to simultaneously achieve sufficient illumination recovery and faithful texture preservation.

To better diagnose this behavior, we qualitatively observe that low-light failure cases are often characterized by flattened intensity histograms and diminished contrast across color channels, which limits the effectiveness of attention-based feature modulation. Although the proposed framework is able to partially recover global brightness, maintaining a satisfactory balance between illumination amplification and fine-detail preservation remains challenging under these adverse conditions. Future work will therefore focus on incorporating illumination-aware modeling strategies to further improve robustness in low-light underwater environments, while effectively mitigating noise amplification during brightness enhancement.

#### 4.3.2. Objective Evaluation

Table 3 reports the quantitative comparison results on the UFO-120, EUVP, and UIEB-90 datasets using both full-reference and non-reference evaluation metrics. Overall, the proposed method achieves competitive or superior performance across most metrics and datasets.

On the UFO-120 dataset, our method achieves the highest PSNR of 29.57 dB and SSIM of 0.945, surpassing both CNN-based methods (e.g., UWCNN and WaterNet) and GAN-based approaches such as UGAN and FUnIE-GAN. Compared with the strongest Transformer-based baseline, URSCT-SESR, our model still yields an improvement of over 2 dB in PSNR and a corresponding increase in SSIM, indicating more accurate structural reconstruction. Meanwhile, the lowest LPIPS score (0.185) confirms that the enhanced images produced by our method are perceptually closer to the reference images. The improvements in non-reference metrics, with UIQM reaching 3.66 and UCIQE 0.598, reflect improvements in colorfulness and contrast.

Similar trends are observed on the EUVP dataset, where our method achieves the best performance in all reported metrics. In particular, the PSNR and SSIM reach 29.35 dB and 0.943, respectively, outperforming URSCT-SESR by a clear margin. The LPIPS score is reduced to 0.152, highlighting the effectiveness of the proposed architecture in preserving perceptually important features under complex real-world underwater conditions. The higher UIQM (3.575) and UCIQE (0.585) scores indicate that our method delivers visually pleasing results with balanced color correction and contrast enhancement, even in scenes with severe spatially varying attenuation.

For the UIEB-90 non-reference dataset, our method also attains the highest UIQM score (3.462) and competitive UCIQE performance (0.621), surpassing most compared methods. Although URSCT-SESR achieves a slightly higher UCIQE value, our method provides a more favorable overall trade-off between color fidelity and perceptual quality, as reflected by the consistently higher UIQM score.

The superior performance across all datasets stems from the collaborative design of our modules. The degradation-aware GAMP module enhances sensitivity to spatial–channel attenuation, the DWMSA bottleneck effectively captures complementary global and local contextual dependencies, and the FARN module further refines texture details and chromatic consistency. Together, these components enable robust underwater image enhancement with consistently improved structural fidelity and perceptual realism.

**Figure 9:**
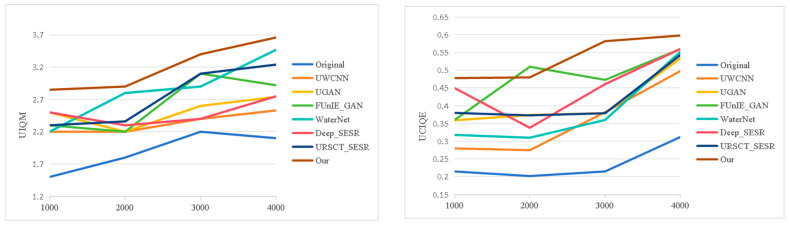
The curves of objective evaluation of various models on the UFO-120.

**Figure 10:**
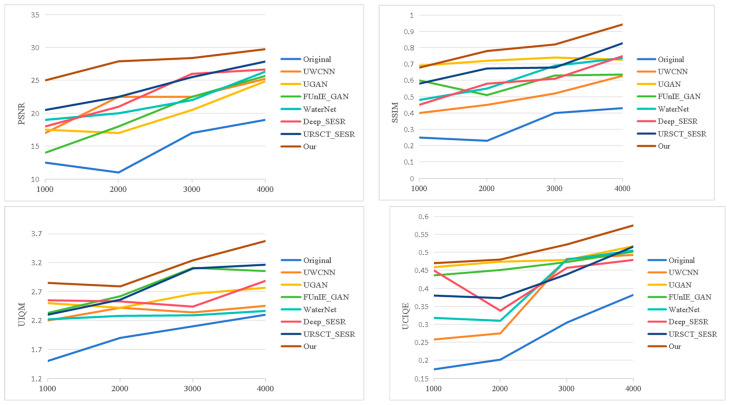
The curves of objective evaluation of various models on the EUVP.

Figure 9 and Figure 10 present the objective evaluation results on the UFO-120 and EUVP datasets, respectively. Our method delivers superior performance over competing approaches. Higher PSNR values indicate more faithful signal reconstruction, whereas SSIM values approaching 1 demonstrate stronger preservation of structural integrity relative to ground truth references. In addition, the proposed model attains leading scores on UIQM and UCIQE, indicating effective color correction and contrast enhancement.

### 4.4. Ablation Experiment

**Figure 11:**
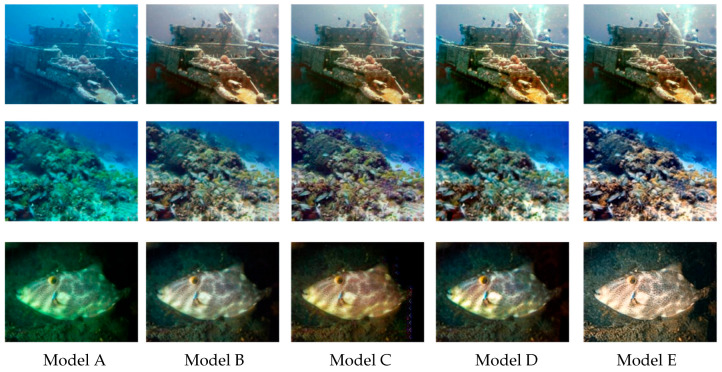
The qualitative performance comparison on the EUVP dataset. From left to right are Baseline, Non-FARN, Non-GAMP, Non-DWMSA, and Our.

To further elucidate the individual and complementary contributions of the DWMSA, GAMP, and FARN modules, ablation experiments were conducted on the EUVP dataset. Several controlled network variants were constructed by removing one module at a time, including Non-FARN, Non-GAMP, and Non-DWMSA, while keeping all other components unchanged. Representative qualitative results are presented in Figure 11. The baseline model, which lacks all three proposed modules, suffers from noticeable color distortion, low contrast, and blurred structural details. When the FARN module is removed (Model B), the restored images exhibit reduced texture sharpness and weakened edge continuity, indicating that adaptive feature refinement contributes to preserving fine-grained details. In the absence of the GAMP module (Model C), the enhanced results show evident color inconsistency and residual chromatic bias, especially in spatially non-uniform regions, highlighting the importance of degradation-aware attention for stabilizing color distribution. Similarly, removing the DWMSA module (Model D) leads to suboptimal global structure coherence and incomplete contrast recovery, suggesting that effective global–local dependency modeling is essential for holistic scene enhancement. By contrast, the Model E integrates all three modules and produces results with improved color balance, clearer structural boundaries, and enhanced contrast.

Table 4 presents the ablation results on the EUVP dataset to evaluate the individual and combined contributions of the proposed DWMSA, GAMP, and FARN modules. Starting from the baseline model (Model A), which only employs the backbone network, the performance is relatively limited, achieving a PSNR of 22.56 dB and an SSIM of 0.756, indicating insufficient capability to handle complex underwater degradations.

By introducing the DWMSA and GAMP modules (Model B), the PSNR improves to 23.24 dB, and the LPIPS is substantially reduced from 0.342 to 0.197, demonstrating that degradation-aware attention and multi-scale feature modeling effectively enhance perceptual similarity. When replacing DWMSA with the FARN module (Model C), further improvements in SSIM (0.776) and non-reference metrics (UIQM 3.087, UCIQE 0.566) are observed, suggesting that adaptive feature refinement plays a key role in improving structural consistency and color quality.

Model D integrates DWMSA and FARN, leading to a notable gain in PSNR (24.46 dB) and SSIM (0.824), which indicates that global–local dependency modeling and feature refinement are complementary. Finally, the full model (Model E), which incorporates all three modules, achieves the best overall performance across all evaluation metrics, with PSNR and SSIM reaching 26.88 dB and 0.896, respectively, and LPIPS reduced to 0.181. The highest UIQM (3.464) and UCIQE (0.589) further confirm superior perceptual quality and color balance.

Overall, the ablation results indicate that each module contributes to performance improvements, supporting the design choices of the proposed network.

Table 5 reports the computational complexity and inference efficiency of different model variants. In addition to FLOPs and parameter counts, actual inference time and frame rate are considered. Model A requires 16.52 G FLOPs and 16.67 M parameters; the introduction of additional modules leads to moderate increases in computational cost.

Model B and Model C exhibit higher complexity due to the inclusion of degradation-aware attention and adaptive feature refinement. Notably, Model D achieves a favorable balance with 18.76 G FLOPs and 18.64 M parameters, suggesting that the DWMSA and FARN modules introduce complementary benefits without excessive computational overhead. The full model (Model E) further refines this balance, requiring only 19.37 G FLOPs and 19.04 M parameters, while delivering the best overall enhancement performance as demonstrated in previous quantitative evaluations. Importantly, under a standard input resolution of 256 × 256 × 3, batch size of 1, and FP16 precision, Model E maintains efficient inference with an average speed of 84.7 FPS on an NVIDIA GeForce RTX 4060, confirming that the additional complexity does not compromise real-time capability.

Overall, the results indicate that the proposed architecture achieves substantial performance gains with a relatively modest increase in computational complexity, suggesting suitability for practical underwater image enhancement scenarios.

Table 6 presents the ablation analysis of the combined loss function on the UIEB dataset. To verify the necessity of each loss component, three controlled experiments were conducted using Model F, Model G. Model H, and Model I.

Table 6 presents the ablation study on the UIEB dataset to evaluate the impact of different loss components, including the chromatic loss $L_{Ch}$, perceptual loss $L_{Per}$, and edge-aware loss $\;L_{Edge}$. When only partial combinations of loss terms are employed (Models F–H), the enhancement performance remains limited, indicating that no single loss component is sufficient to fully address the diverse degradation characteristics of underwater images.

Model F, which combines $L_{Ch}$ and $L_{Per}$, achieves moderate improvements in SSIM (0.765) and LPIPS (0.192), suggesting that chromatic consistency and perceptual alignment contribute to improved visual similarity. Model G, incorporating $L_{Per}$ and $L_{Edge}$, exhibits slightly better UCIQE (0.537), reflecting enhanced edge contrast but reduced structural stability, as indicated by the lower SSIM. In contrast, Model H, which integrates $L_{Ch}$ and $L_{Edge}$, yields more balanced performance across both full-reference and non-reference metrics, achieving higher PSNR (23.21 dB) and UIQM (3.087).

The best performance is obtained by Model I, which jointly employs all three loss terms. This full loss configuration boosts PSNR to 25.79 dB and SSIM to 0.856, while reducing LPIPS to 0.186. Moreover, the highest UIQM (3.358) and UCIQE (0.594) scores demonstrate improved color fidelity, edge clarity, and perceptual quality. These results confirm that the proposed combined loss function provides complementary supervision signals, leading to more stable optimization and superior enhancement performance.

## 5. Conclusions

Underwater imagery is fundamentally impaired by complex degradations, ranging from contrast suppression and severe color shifts to noise accumulation and structural blurring. To confront these challenges, we present an end-to-end enhancement framework built upon a perceptual Vision Swin Transformer backbone. The architecture integrates three purpose-designed components: a DWMSA module that jointly captures long-range dependencies and fine-grained local cues; a GAMP module that adaptively emphasizes spatially and chromatically degraded regions; and a FARN that alleviates gradient degradation while reinforcing texture fidelity and color accuracy.

Extensive quantitative evaluations across multiple benchmark datasets demonstrate that the proposed model consistently outperforms state-of-the-art methods in terms of image quality metrics, while subjective visual comparisons further confirm notable improvements in clarity, color balance, and structural realism. In addition, efficiency analysis under a standard input resolution shows that the proposed framework maintains real-time inference capability, indicating a favorable trade-off between restoration performance and computational cost.

Despite strong overall performance, the model exhibits limitations in low-light underwater environments. As illustrated by the reported failure cases, severe absorption and scattering in such scenarios significantly suppress global illumination and obscure fine structural details, leading to pronounced chromatic distortion. While the proposed framework can partially recover illumination, balancing brightness amplification and detail preservation remains challenging. Future efforts will explore illumination-aware learning strategies, enhanced task-driven evaluation protocols, and more specialized loss formulations to better accommodate severely compromised imaging conditions. Furthermore, we plan to extend the framework to real-world applications—including marine ecosystem monitoring, underwater robotics, video enhancement, and AI-assisted perception—to broaden its practical impact and validate its utility in operational settings.

## Figures and Tables

**Table 1:** Experimental and implementation parameters used for training and evaluation.

Parameter	Value
GPU	NVIDIA GeForce RTX 4060 (8 GB VRAM)
Framework	PyTorch 2.1.0
RAM	32 GB
CUDA & cuDNN	CUDA 11.8
Optimizer	Adam
Learning rate	0.0001
Batch size	8

**Table 2:** Average subjective visual quality scores evaluated by 50 human observers.

Method	UWCNN	UGAN	FUnIE_GAN	WaterNet	Deep_SESR	URSCT_SESR	Our
Mean	4.562	5.352	6.853	7.027	7.572	7.548	8.539

**Table 3:** The results of objective evaluation for different methods on UFO-120, EUVP and UIEB-90.

Dataset	Method	UWCNN	UGAN	FUnIE_GAN	WaterNet	Deep_SESR	URSCT_SESR	Our
UFO-120	PSNR	25.43	24.34	26.73	23.86	26.82	27.55	29.57
	SSIM	0.723	0.744	0.685	0.733	0.694	0.862	0.945
	LPIPS	0.342	0.215	0.312	0.354	0.257	0.253	0.185
	UIQM	2.53	2.74	2.92	2.47	2.75	3.24	3.66
	UCIQE	0.498	0.534	0.56	0.552	0.56	0.543	0.598
EUVP	PSNR	25.24	24.87	25.68	26.34	26.66	27.87	29.35
	SSIM	0.628	0.727	0.636	0.787	0.749	0.828	0.943
	LPIPS	0.316	0.193	0.286	0.315	0.301	0.273	0.152
	UIQM	2.454	2.766	3.054	2.365	2.886	3.164	3.575
	UCIQE	0.513	0.517	0.502	0.505	0.479	0.516	0.585
UIEB-90	UIQM	2.462	2.256	2.856	2.743	2.953	3.068	3.462
	UCIQE	0.521	0.538	0.527	0.527	0.563	0.624	0.621

**Table 4:** Ablation analysis of network structure based on the EUVP dataset. “√” denotes that the module is selected (enabled), and “×” denotes that the module is removed (disabled).

	BASELINE	DWMSA	GAMP	FARN	PSNR	SSIM	LPIPS	UIQM	UCIQE
Model A	√	×	×	×	22.56	0.756	0.342	2.964	0.503
Model B	×	√	√	×	23.24	0.753	0.197	2.975	0.549
Model C	×	×	√	√	23.36	0.776	0.205	3.087	0.566
Model D	×	√	×	√	24.46	0.824	0.210	3.124	0.541
Model E	×	√	√	√	26.88	0.896	0.181	3.464	0.589

**Table 5:** Analysis of computational complexity.

Model	FLOPs/G	Parameters/M	Inference Time/ms	FPS
Model A	16.52	16.67	9.6	104.2
Model B	20.26	18.48	13.8	72.5
Model C	21.33	19.79	14.6	68.5
Model D	18.76	18.64	12.1	82.6
Model E	19.37	19.04	11.8	84.7

**Table 6:** Ablation analysis of combined loss function on UIEB dataset. “√” denotes that the module is selected (enabled), and “×” denotes that the module is removed (disabled).

	$L_{Ch}$	$L_{Per}$	$L_{Edge}$	PSNR	SSIM	LPIPS	UIQM	UCIQE
Model F	√	√	×	22.32	0.765	0.192	2.989	0.525
Model G	×	√	√	22.52	0.736	0.210	2.983	0.537
Model H	√	×	√	23.21	0.785	0.195	3.087	0.542
Model I	√	√	√	25.79	0.856	0.186	3.358	0.594

## Data Availability

The original contributions presented in this study are included in the article. Further inquiries can be directed to the corresponding author.
